# Autoinsertion of soluble oligomers of Alzheimer's Aβ(1–42) peptide into cholesterol-containing membranes is accompanied by relocation of the sterol towards the bilayer surface

**DOI:** 10.1186/1472-6807-6-21

**Published:** 2006-10-19

**Authors:** Richard H Ashley, Thad A Harroun, Thomas Hauss, Kieran C Breen, Jeremy P Bradshaw

**Affiliations:** 1 Biomedical Sciences, College of Medicine and Veterinary Medicine, University of Edinburgh, Edinburgh EH8 9XD, UK; 2 Canadian Neutron Beam Centre, Chalk River Laboratories, Chalk River, Ontario K0J 1J0, Canada; 3 Abteilung SF2/Biophysik, Hahn-Meitner-Institut, D-14109 Berlin, Germany, and Physical Biochemistry, Dept. of Chemistry, DarmstadtUniversity of Technology, Petersenstrasse 22, D-64287 Darmstadt, Germany; 4 Department of Psychiatry, Ninewells Hospital and Medical School, University of Dundee, Dundee DD1 9SY, UK; 5 Veterinary Biomedical Sciences, College of Medicine and Veterinary Medicine, University of Edinburgh, Edinburgh EH25 9RG, UK; 6 Department of Physics, Brock University, St. Catharines, Ontario L2S 3A1, Canada; 7 The Parkinson's Disease Society of the United Kingdom, London SW1V 1EJ, UK

## Abstract

**Background:**

Soluble Alzheimer's Aβ oligomers autoinsert into neuronal cell membranes, contributing to the pathology of Alzheimer's Disease (AD), and elevated serum cholesterol is a risk factor for AD, but the reason is unknown. We investigated potential connections between these two observations at the membrane level by testing the hypothesis that Aβ(1–42) relocates membrane cholesterol.

**Results:**

Oligomers of Aβ(1–42), but not the monomeric peptide, inserted into cholesterol-containing phosphatidylcholine monolayers with an anomalously low molecular insertion area, suggesting concurrent lipid rearrangement. Membrane neutron diffraction, including isomorphous replacement of specific lipid hydrogens with highly-scattering deuterium, showed that Aβ(1–42) insertion was accompanied by outward displacement of membrane cholesterol, towards the polar surfaces of the bilayer. Changes in the generalised polarisation of laurdan confirmed that the structural changes were associated with a functional alteration in membrane lipid order.

**Conclusion:**

Cholesterol is known to regulate membrane lipid order, and this can affect a wide range of membrane mechanisms, including intercellular signalling. Previously unrecognised Aβ-dependent rearrangement of the membrane sterol could have an important role in AD.

## Background

Alzheimer's Disease (AD) is associated with neurofibrillary tangles containing tau proteins, and with extracellular amyloid plaques containing fibrils formed by Aβ(1–40), Aβ(1–42) and Aβ(1–43), amyloid-β (Aβ) peptides generated by intracellular proteolytic cleavage of Amyloid Precursor Protein (APP), specifically the neuronal membrane APP homologue, APLP-1. Although Aβ peptides, especially Aβ(1–42), are known to play an active role in the development of AD [1], recent observations suggest that extracellular Aβ fibrils and plaques are relatively inert [2], like other amyloid fibrils (e.g. those formed by Islet Amyloid Polypeptide, IAPP [3]), raising questions about the precise mechanism of Aβ peptides.

Attention has recently become focussed on pre-fibrillar Aβ assemblies, especially globular oligomers [4-6] normally containing from 2 to 6 molecules. Unlike mature fibrils, Aβ oligomers are soluble and "membrane-active". They may be crucial to the toxicity of Aβ because they can form specific ion channels, or disrupt and permeabilise membranes through less well-defined mechanisms [7]. Interestingly, different Aβ peptides form specific, soluble oligomers in diverse ways [6], which could help to explain their individual "toxicities", and although early studies suggested that monomeric Aβ, as opposed to oligomeric Aβ, formed discrete ion channels [8], the active species may well have been a (lipid-promoted) oligomer [9]. In terms of a testable theory to explain the mechanism of AD, membrane permeabilisation by membrane-active forms of Aβ could compromise cell function and promote cell death [10].

Although the precise role of Aβ oligomers in AD remains speculative, their discovery calls for caution in the use of plaque-disrupting anti-Aβ antibodies [11], because increasing the relative levels of membrane-active forms of Aβ might in fact accelerate the disease. These recent findings have also highlighted significant gaps in our understanding of the structural basis of the interactions of Aβ peptides with cell membranes, including the importance of individual membrane lipids, especially cholesterol. Cholesterol is a well-established risk factor in AD, but its role is complex, and many of its disease-related activities appear to be metabolic [12] rather than structural. However, a plasma-membrane like concentration of cholesterol (30 mol%) promoted Aβ(1–40) insertion and channel formation in planar lipid bilayers [13], suggesting the (non-esterified) sterol (as opposed to total cholesterol, including circulating cholesterol esters) may be directly implicated in the pathological mechanism of Aβ at the level of the cell membrane. We set out to test the idea that well-defined oligomers [4] of Aβ(1–42) can insert into cholesterol-containing membranes, and promote changes in membrane structure.

Our first objective was to establish that oligomeric Aβ(1–42) inserts into membranes, and to confirm that (as predicted) monomeric Aβ(1–42) does not. Having found that cholesterol promoted the insertion of Aβ oligomers into phosphatidylcholine monolayers, with a paradoxically small molecular insertion area suggesting concurrent lipid rearrangement, we used lamellar neutron diffraction to locate the individual membrane components. We discovered that peptide insertion is associated with significant displacement of the sterol towards the polar surfaces of the bilayer, leading to a measurable effect on membrane lipid order.

## Results

### Autoinsertion of Aβ oligomers into membranes

**Figure 1:**
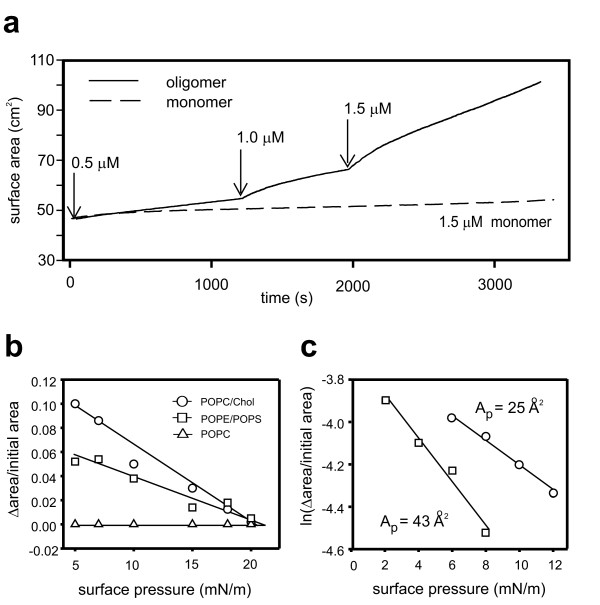
**Insertion of Aβ(1–42) oligomers into lipid monolayers**. Panel a illustrates spontaneous insertion of oligomeric but not monomeric Aβ(1–42) into a monolayer containing 7:3 (mol/mol) POPC:Chol compresssed to a lateral surface pressure of 15 mN/m (representative of 6 similar experiments). Panel b shows exclusion of peptide oligomers at a surface pressure of ~20 mN/m in binary lipid mixtures containing POPE/POPS or POPC/Chol. Note the lack of insertion in POPC monolayers without Chol. Panel c shows the molecular insertion areas of the peptide oligomer. All the points are means of 2 experiments, and the molar concentrations of the peptide refer to the initial monomer concentrations.

Monolayers (Langmuir-Blodgett films) provide a useful *in vitro *model system to investigate membrane/protein interactions [14,15]. We noted that oligomeric Aβ(1–42), but not monomeric Aβ(1–42), inserted spontaneously into mixed chain palmitoyl-oleoyl phosphatidylcholine (POPC) monolayers containing cholesterol (Chol) (Fig. 1a), with an exclusion pressure of ~20 mN/m (Fig. 1b). Insertion was inhibited by 5 μM rifampicin, as shown previously for IAPP oligomers [16]. The oligomers also inserted into negatively charged PO-phosphatidylethanolamine/PO-phosphatidylserine monolayers, but not into POPC monolayers in the absence of cholesterol, consistent with the lack of Aβ channel activity in POPC planar bilayers [13].

The oligomers had an unexpectedly small ''molecular insertion area'' (Fig. 1c), especially in POPC/Chol, lower than the minimum cross-sectional area of a single alpha helix (~100 Å^2^). This suggested that protein insertion was accompanied by changes in lipid packing, with a net reduction in lipid surface area, and we proceeded to examine the structure of Aβ/cholesterol-containing lipid bilayers using membrane neutron diffraction.

### Lipid profiles derived from lamellar neutron diffraction data

Unlike X-rays, cold neutrons are well tolerated by biological materials, and cause relatively little structural damage. Taking advantage of this, neutron diffraction measurements were carried out on multibilayer lipid samples enclosed in airtight humidified chambers, with and without pre-incorporated oligomeric protein. The lamellar D-repeat of 7:3 (mol/mol) POPC:Chol multibilayers, calculated using the Bragg equation from 5 orders of diffraction at a relative humidity of 93.6%, was 57.2 ± 0.97 Å (mean ± SD, n = 3). This was slightly reduced following the incorporation of Aβ(1–42) oligomers, to 56.5 ± 0.42 Å (mean ± SD, n = 6). However, the difference is not significant. Observations over 24 hours confirmed that the diffraction peak positions and intensities (areas) did not alter with time, indicating the samples were fully equilibrated, and remained structurally stable.

**Figure 2:**
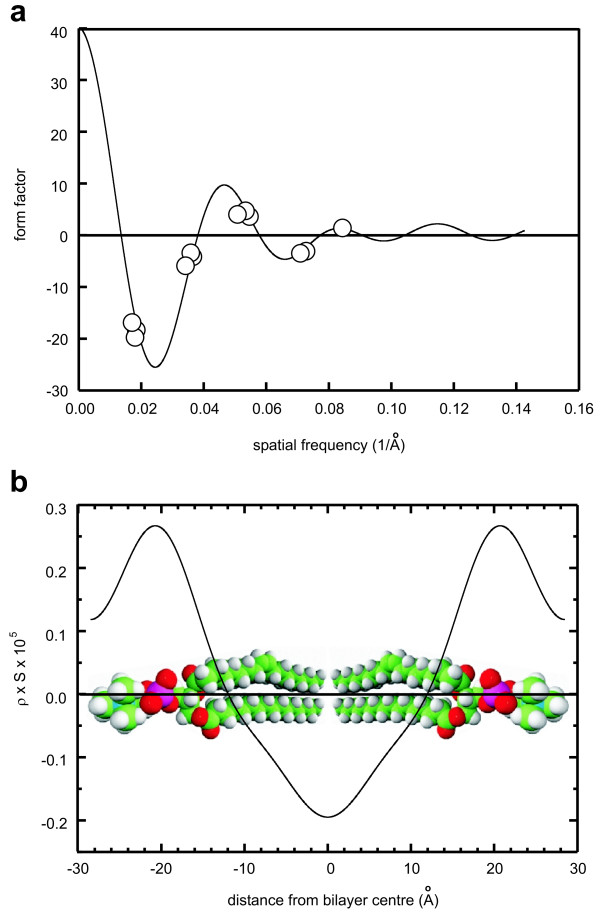
**Calculation of bilayer scattering length density profiles from neutron diffraction data**. Panel a shows structure factors obtained from POPC/Chol (70:30 mol%) membranes, and fitting to a continuous transform for phase determination (- - + - + for orders #1–5 in this example). The data points (open circles) include measurements at up to 3 different relative humidities (i.e. 3 different lamellar spacings). Panel b shows the scattering length density (SLD) profile calculated by Fourier synthesis using the signed form factors obtained in part a, after scaling the data to match the mean scattering length of the bilayer unit cell. The main features of the centrosymmetric profile, corresponding to the space-filling model of POPC, are described in detail in the text.

To help phase the diffraction data, and obtain more detailed structural information, the lamellar D-repeat was systematically varied by changing the membrane hydration. The D_2_O/H_2_O ratio was also varied in samples with and without incorporated peptide, to determine the water profile across the bilayer. Fig. 2 illustrates how the data were fitted to assign signs (phases) to structure factors derived from diffraction peak intensities. In Fig. 2a, unscaled structure factors for 5 orders of diffraction at up to 3 different relative humidities (i.e. up to 3 different lamellar spacings) were plotted in reciprocal space and systematically assigned positive or negative values to achieve the best least-squares fit to a smooth continuous transform, and thereby satisfy Shannon's sampling theorem. The phased structure factors were then placed on a relative absolute scale, and the scattering length density profile (SLD) shown in Fig. 2b was derived by Fourier synthesis.

As illustrated by the space-filling POPC molecular models in part b, the lowest point of the profile, with negative scattering, marks the exact centre of the bilayer, corresponding to the hydrogen-rich terminal methyl groups of the POPC acyl chains. The peaks of positive neutron scattering are associated with the phosphoester region of POPC, situated towards the polar surfaces of the bilayer. The centrosymmetric "kinks" in the profile, ~10 Å from the midpoint, reflect the oleoyl acyl chain double bonds. A table of all the experimentally derived structure factors used to calculate transbilayer SLD profiles in the presence of 8.06 mol% D_2_O, including the form factor errors from the fitting procedure (see Methods), is provided in Additional File 1.

### Aβ oligomers displace membrane cholesterol

We next carried out experiments to locate the two membrane lipids, POPC and Chol, in the presence and absence of 1 mol% peptide, incorporated as pre-formed oligomers. Appropriate neutron diffraction data were phased and placed on a relative absolute scale, and SLD profiles were constructed by Fourier synthesis. Difference (subtraction) profiles revealed the transbilayer distribution of specific membrane components, including components in which specific hydrogen atoms were isomorphously replaced by deuterium. As previously noted [17], 4 orders of diffraction were sufficient to achieve optimum (sub-Å) resolution of the specifically labelled components, and the mean positions of the labels were well-defined by symmetrical, single-component Gaussian distributions in each leaflet of the centrosymmetric bilayer.

**Figure 3:**
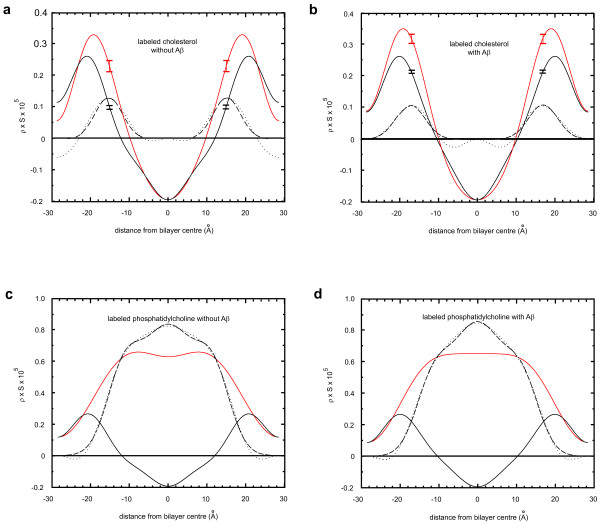
**Displacement of membrane cholesterol by Aβ(1–42) oligomers**. Panels a and b illustrate how the positions of the labels in deuterated Chol were determined, in the presence and absence of Aβ(1–42), respectively. The solid black lines are the SLD profiles obtained with unlabelled Chol, and the solid red lines are the profiles with labelled Chol. The dotted lines are the corresponding difference (plus or minus peptide) profiles, and the dashed lines are best-fit single Gaussian fits to these difference profiles, showing peaks at 15.11 ± 0.39 Å from the bilayer centre (without peptide), and 17.06 ± 0.26 Å from the bilayer centre (with peptide), respectively (mean mid-peak positions ± SD). The errors bars show the 95% confidence intervals of the SLD profiles at the mid-difference profile positions. Panels c and d show similar difference profiles and fits without and with membrane Aβ(1–42) for the uniformly-labelled palmitoyl acyl chains of POPC, with similar figure labelling.

The location of membrane cholesterol was determined by collecting and analysing data from membranes in which the lipid was either unlabelled (protonated), or deuterated on its planar sterol rings. The resulting difference profile (Fig. 3a) was compared to a difference profile from samples containing Aβ(1–42) oligomers (Fig. 3b). Strikingly, in the presence of the peptide, membrane cholesterol was displaced towards the surface of the bilayer, by 2.0 Å in each leaflet. The 95% confidence limits of the experimental profiles (shown for regions corresponding to the maxima of the two difference peaks) were well separated, and the calculated fits showed a highly significant difference in the position of membrane cholesterol with and without the peptide (P < 0.001). In contrast, the position of the (labelled) phospholipid acyl chains was only slightly altered, in the opposite direction (Fig. 3, parts c and d).

### The membrane locations of Aβ oligomers and water

**Figure 4:**
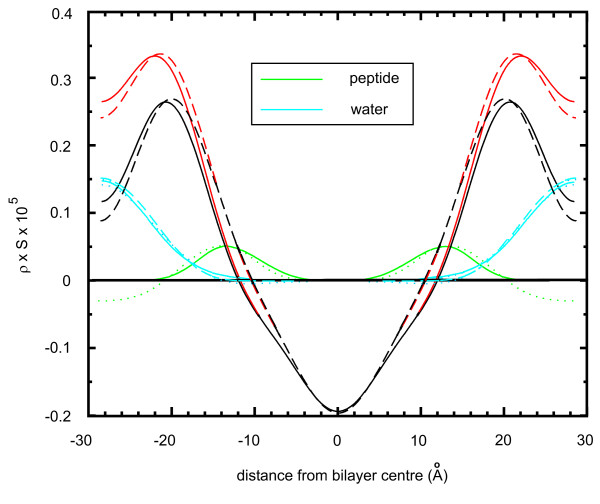
**Intramembrane positioning of Aβ(1–42) oligomers, and the distribution of membrane water**. The peptide was localised (dotted green line) by subtracting profiles obtained in 8.06 mol% D_2_O in the presence and absence of the peptide (dashed and solid black lines, respectively). The difference profile was fitted to 2 Gaussian distributions (solid green lines), as described for Fig. 3. The water difference profiles (dotted blue lines) were constructed by subtracting profiles obtained in 8.06 mol% D_2_O in the presence and absence of peptide, from profiles of the same samples in 20 mol% D_2_O (dashed and solid red lines, respectively). The water distributions are fitted to single Gaussians (dashed and solid blue lines, with maxima at the edges of the profiles). Note the substantial overlap in the presence and absence of peptide.

A similar difference Fourier approach was used to locate the peptide oligomer, and membrane-associated water, in bilayers containing cholesterol (Fig. 4). Two centres of mass were apparent for the unlabelled peptide oligomers, each 13.0 Å from the centre of the bilayer, consistent with deep penetration of the peptide into each leaflet of the membrane. Transbilayer water distributions were revealed by comparing the SLD profiles of paired samples, with or without incorporated peptide, between 8.06 mol% D_2_O, where the water is effectively "invisible" to neutrons, and 20 mol% D_2_O, where it diffracts strongly. Unlike IAPP) [18], the incorporation of Aβ oligomers was not associated with a pore-like water column extending all the way through the bilayer, and the water profiles were very similar in the presence and absence of the peptide (Fig. 4).

### Membrane lipid order

**Figure 5:**
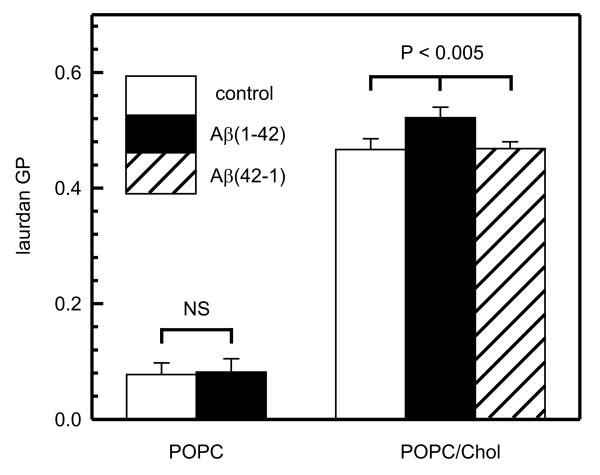
**Modification of membrane lipid order by Aβ(1–42) oligomers**. Lipid order as reported by laurdan GP analysis ("laurdan GP") is low in POPC liposomes reconstituted with or without the peptide, but the GP values are much higher in the presence of 30 mol% cholesterol, consistent with phospholipids in mixed liquid ordered (Lo) and liquid disordered (Ld) phases. The disorder decreases significantly in liposomes reconstituted in the presence of Aβ(1–42) but not Aβ(42–1). All the data are means ± SD of 6 independent experiments carried out at 25°C.

The relatively rigid, planar rings of membrane cholesterol intercalate between the flexible acyl chains of neighbouring phospholipids, making the sterol a key determinant of membrane lipid order in eukaryotic cell membranes. To determine whether the outward displacement of cholesterol affects membrane lipid order, as predicted, we calculated the generalised polarisation (GP) of laurdan, which undergoes a water dipole-dependent shift in its emission spectrum that can be directly related to lipid order. [19,20]. At 25°C, membrane POPC was highly disordered, as expected, with a very low mean GP (Fig. 5). The value was unchanged in liposomes co-reconstituted with 1 mol% Aβ oligomers, which do not insert into POPC membranes in the absence of cholesterol (Fig. 1b). The GP value for POPC/Chol liposomes was much higher, reflecting the appearance of an ordered, cholesterol-mediated, Lo phospholipid phase. The incorporation of Aβ(1–42) was accompanied by a significant increase in lipid order, whereas co-reconstitution of liposomes with the non-membrane active reverse peptide, Aβ (42–1), had no effect.

## Discussion

### Experimental materials

We reconstituted Aβ(1–42) rather than partial-length peptides, to enhance the relevance of our *in vitro *structural studies to the interaction of Aβ peptides with cholesterol-containing membranes *in vivo*. We also avoided mixing lipids and proteins in non-physiological organic solvents, and prepared membrane-active water-soluble oligomers, as described in the Background to this report. Finally, we used POPC, containing one saturated and one mono-unsaturated acyl chain. POPC has a main chain melting (gel to liquid-crystalline phase transition) temperature close to 0°C, well below the experimental temperature of 25°C, but because it is a synthetic mixed-chain phospholipid, it cannot physically separate into complex 2-component mixtures containing saturated and unsaturated phospholipids. However, it still allows membrane cholesterol to intercalate normally in the bilayer [21].

Many previous structural studies have been carried out on the amyloidogenic Aβ fragment Aβ(25–35), including membrane-based studies involving (small angle) X-ray diffraction [22] and neutron diffraction [23]. The fragment can be incorporated into both neutral and negatively-charged bilayers, and like Aβ(1–42) it is located within the hydrophobic, acyl-chain region. The peptide was exposed to lipid membranes for prolonged periods of up to 90 min, and could have become incorporated as an oligomer or as a monomer. Aβ(1–40), a physiological form of Aβ, has also previously been incorporated into monolayers [24]. The membranes had to be negatively charged, as in the present study in the absence of cholesterol, and the exclusion pressure was between 20–32 mN/m, also similar to the present study, again suggesting that spontaneous insertion requires relatively "loose" lipid packing. Interestingly, the Aβ(1–40) solution was equilibrated for 1–2 hours in a low-ionic strength buffer at room temperature. Aβ(1–40) comprises a mixture of monomers and oligomers [6], and incubation for 1–2 hours will have encouraged polymerisation [4].

### Membrane autoinsertion of Aβ(1–42) oligomers

Aβ(1–42) oligomers (but not monomers) incorporated spontaneously into POPC monolayers containing cholesterol, but not into pure POPC monolayers. Their paradoxically small molecular insertion area (< 100 Å^2^) suggested concomitant lipid rearrangement, and membrane neutron diffraction showed that peptide insertion was accompanied by significant outward displacement of membrane cholesterol as the peptide was accommodated between the POPC acyl chains. Lamellar diffraction does not probe structures in the plane of the bilayer, and any expansion of the unit cell in this direction would remain undetectable on the ''per lipid'' scale we adopted. However, despite our inability to see lateral reorganisation due to peptide insertion, we noted slight membrane thinning in the presence of the peptide (compare Fig. 3c to Fig. 3d), consistent with the idea that the protein is accommodated between the phospholipid acyl chains. This reflects the conservation of lipid volume. Briefly, following insertion of a peptide taking up potential ''lipid headgroup'' space, a larger ''effective surface area'' per lipid will require a reduction in length, and shift the centre of mass towards the centre of the unit cell.

Our study does not provide detailed information about the structure of membrane-located Aβ oligomers, other than to suggest that (because of their position) the local environment is highly non-polar. NMR structures of monomeric Aβ(1–42) in an apolar solvent revealed a hairpin-like molecule containing two α-helices between residues 8–25 and 28–38, respectively [25]. However, the conformations of the Aβ peptide in oligomers may differ substantially, and further insights may only be possible when the solution or membrane structures of the membrane-active oligomers become available. The absence of a contiguous "transbilayer" water profile (*cf*[18]) does not entirely exclude the possibility of proteineaceous transmembrane channels, which may be mostly closed in our conditions. The discrete peptide peaks may, however, argue against a conventional transmembrane protein pore.

At 25°C, POPC membranes containing 30 mol% cholesterol adopt a mixture of fluid, liquid-ordered (Lo) and liquid disordered (Ld) phases [26], and the sterol is laterally well-dispersed [21]. In our study, in the absence of Aβ, the centre of mass of (labelled) membrane cholesterol (15.1 Å from the centre of the bilayer in each membrane leaflet) places its hydroxyl group 17.0 Å from the bilayer centre, just into the hydrophilic region of the bilayer [27]. This is consistent with promotion of the Lo phase. Outward displacement of the sterol by 2 Å in the presence of Aβ(1–42) oligomers increased membrane lipid order, consistent with the effect of Aβ(1–40) on membrane lipid order previously reported by the steady-state anisotropy of diphenylhexatriene in cholesterol-containing liposomes [28], which also required aggregated forms of Aβ [29]. "Vertical" (as opposed to lateral) relocation of membrane cholesterol, even to the extent of "extruding" the sterol [30], together with accompanying changes in membrane lipid order, could have an important impact on cell membranes [31], affecting for example receptors [32], signal transduction systems [33] and endocytosis [34].

### Aβ(1–42) oligomers and cholesterol as risk factors for AD

Kuo et al. [35] noted over 10 years ago that the cerebral cortices of brains from patients with AD contained a large excess of water-soluble forms of Aβ(1–40) and Aβ(1–42), including monomeric and oligomeric forms of the peptides. In primary human neurones, dimers actually appeared to form within the cell itself [36], and in experiments on animals, dimers and trimers were shown to inhibit synaptic long-term potentiation (LTP) *in vivo *[37]. LTP is believed to be a key molecular process underlying some forms of learning and memory, and this important modification was mainly caused by trimers [38]. Dimers and tetramers are less effective [38], and memory deficits in Tg2576 mice (transgenic animals that express a human AD-associated APP variant) have recently been attributed to oligomers containing up to 12 peptides [39]. In line with all these findings, localisation studies (e.g. [40] have supported the idea that oligomeric forms of Aβ preferentially associate with synaptic membranes.

Cholesterol affects the production of Aβ peptides, consistent with its multiple, complex roles in cells [12], but increased levels of the peptides appear to be associated with low, not high, membrane cholesterol [41], as seen for example with the reduced membrane cholesterol:phospholipid ratio in *post-mortem *brain samples from patients with AD [42]. Cholesterol-deficient cell membranes are also more susceptible to the destabilizing effects of Aβ [10]. These observations appear to suggest that increasing the amount of the membrane sterol ought to protect against AD, and increased serum cholesterol may be a risk factor for completely unrelated reasons [41]. Yip et al. [43] applied fibrillar and oligomeric forms of Aβ(1–40) to NGF-differentiated PC12 cells, and found that adding cholesterol reduced cell surface Aβ staining, whereas depleting membrane cholesterol (using cyclodextrin) increased staining. The authors suggested that elevated (membrane) cholesterol is a risk factor for AD because it increases membrane lipid order, which in turn inhibits the interaction of Aβ with the membrane, leaving more of the peptide free in the extracellular space to form potentially damaging fibrils.

Our structural study has focussed on oligomeric Aβ(1–42) rather than fibrillar Aβ, and we have demonstrated that membrane cholesterol relocates towards the bilayer surface following spontaneous incorporation of the peptide. This is consistent with the known toxicity of Aβ oligomers, and also with the previous observations of Yip et al. [43], where Aβ oligomers and membrane cholesterol appeared to "compete" for a similar membrane location. Relocation of the free sterol following the autoinsertion of Aβ(1–42) oligomers could affect many important membrane processes both directly and indirectly (e.g. by changing membrane lipid order), and we suggest that the transbilayer location of membrane cholesterol may be a key factor in AD.

## Conclusion

Oligomeric Aβ(1–42) autoinserts spontaneously into cholesterol-containing membranes, and insertion is accompanied by striking outward displacement of the sterol by 2 Å in each membrane leaflet, leading to functional alterations in membrane lipid order. Our findings suggest that in patients with AD, the specific membrane location of cholesterol may be more important than the absolute amount. Even if membrane cholesterol is reduced in AD (perhaps because it is displaced or extruded by Aβ oligomers), any increase in the amount that remains (induced, for example, by an elevated serum cholesterol concentration) could have a very marked effect on neuronal cell function, because of the unusual location of the lipid.

## Methods

### Materials

POPC, POPC labelled with deuterons on the palmitoyl chain, PO-phosphatidylethanolamine (POPE), PO-phosphatidylserine (POPS) and cholesterol were purchased from Avanti Polar Lipids (AL, USA). Chol containing 6 non-acyl deuterons ([2,2,3,4,4,6-^2^H_6_) cholesterol) was obtained from CDN Isotopes (Pointe-Claire, Quebec, Canada). Laurdan (6-dodecanoyl-2-dimethylaminonaphthalene), from Molecular Probes Europe (Invitrogen, Paisley, UK), was a kind gift from Rory Duncan (University of Edinburgh). Alzheimer's Aβ(1–42) and Aβ(42–1) peptides were synthesised by Biopeptide (San Diego), and Aβ(1–42) oligomers were prepared and separated from fibrillar Aβ as detailed previously [4] (Additional File 2.). Protein concentrations were determined by the micro Bio-Rad procedure, using Aβ(1–42) standards.

### Langmuir-Blodgett (LB) films

LB films (monolayers) were formed as previously described [16] in a Nima Technology trough (Coventry, UK), and experiments were carried out under both constant area and constant pressure conditions. Exclusion pressures were determined by clamping the surface area at different initial surface pressures (π, mN/m), and measuring the change in π over 200 s. Peptide molecular insertion areas (A_p_) were determined by clamping π and measuring the relative change in monolayer area (ΔA_m_) over 200 s. Given that ΔA_m _= A_m_.exp(-A_p_π/*k*T), ln(ΔA_m_/A_m_) = -A_p_/*k*Tπ, so that the slopes of suitable plots provided -A_p_/*k*T (where *k*T is 4.2 × 10^-21 ^Joules).

### Sample preparation for neutron diffraction

POPC and Chol were weighed in glass tubes and dispersed in chloroform at a total lipid concentration of 50 mg/ml and a molar ratio of 7:3, respectively. The chloroform was removed by drying under a stream of N_2 _to leave a thin lipid film, and drying was completed under vacuum for at least 12 hrs. Aβ(1–42) oligomers (450 μg/ml), prepared as described above, were dialysed for 2–3 hours against ultrapure water containing 20% (w/v) PEG 32K (adjusted to a pH of 7.4 with a trace of KOH), to reduce the projected final sample volume to 0.5 ml. The dried lipid films were re-wetted in pure water or a solution of Aβ oligomers (1 mol%, calculated as the original monomer concentration), and the components were mixed by bath sonication under N_2 _until the lipid film had been completely resuspended to produce a turbid, multilamellar, proteolipid vesicle suspension. This was then applied to a clean silicon crystal substrate, allowed to dry undisturbed in air to avoid the formation of air bubbles, and finally dried to completion under vacuum. The dried multibilayers were mounted in sealed sample cans immediately before use, and rehydrated *in situ *as described below.

### Neutron data collection

Lamellar diffraction measurements were carried out on membrane diffractometer N5 at the Canadian Neutron Beam Centre (CNBC), Chalk River Laboratories, Ontario, Canada, using a wavelength of 2.37 Å. Samples containing 10 mg lipid were thermostatically maintained at 25°C in the presence of saturated solutions of KCl, KNO_3 _or K_2_SO_4 _in 8.06 mol% or 20 mol% D_2_O. The salt solutions set the relative humidity to 84.3%, 93.6% or 97.3%, respectively. We ensured that the entire sample remained vertical and completely within the beam as the sample holder was rotated by *θ *degrees and the detector was scanned around 2*θ *to record Bragg peaks. Data corresponding to 1–5 orders of diffraction were collected with counting errors below 1%, and corrected for variations in beam intensity.

### Neutron data analysis

After fitting and subtracting the backgrounds around the Bragg reflections, peak areas (intensities,* I*) were fitted to Gaussian distributions using PeakFit (SPSS), and the lamellar repeat distance (Bragg spacing, *d*) was calculated by least squares fitting the midpoints of the peaks to the Bragg equation: n*λ *= 2*d*.sin*θ*_*n*_, for orders *n *= 1 to 5. Structure factor amplitudes (SFA) were calculated by square-rooting the corrected peak intensities: |F(q)|=CabsClorI
 MathType@MTEF@5@5@+=feaafiart1ev1aaatCvAUfKttLearuWrP9MDH5MBPbIqV92AaeXatLxBI9gBaebbnrfifHhDYfgasaacH8akY=wiFfYdH8Gipec8Eeeu0xXdbba9frFj0=OqFfea0dXdd9vqai=hGuQ8kuc9pgc9s8qqaq=dirpe0xb9q8qiLsFr0=vr0=vr0dc8meaabaqaciaacaGaaeqabaqabeGadaaakeaadaabdaqaaiabdAeagjabcIcaOiabdghaXjabcMcaPaGaay5bSlaawIa7aiabg2da9maakaaabaGaem4qam0aaSbaaSqaaiabdggaHjabdkgaIjabdohaZbqabaGccqWGdbWqdaWgaaWcbaGaemiBaWMaem4Ba8MaemOCaihabeaakiabdMeajbWcbeaaaaa@4102@. The correction factor *C*_*abs *_= *α*/(1–*e*^-*α*^) was applied to account for neutron absorption (*α *= 2 *μz*/sin*θ*, where *μ* is the calculated absorption coefficient and *z *is the sample thickness) and the geometric Lorentz correction factor *C*_*lor *_= sin2*θ *was applied to compensate for the fixed axis of rotation of the sample.

8.06 mol% D_2_O does not contribute to the diffraction pattern (because at this molar ratio the negative scattering of hydrogen nuclei is exactly balanced by the positive scattering of oxygen and deuterium nuclei), so structure factors obtained from samples in 8.06 mol% D_2_O at 3 different relative humidities (i.e. with 3 different lamellar repeats) were phased directly in reciprocal space by reconstructing the continuous form factor curve using Shannon's sampling theorem. The procedure was carried out as previously described [44], simultaneously least squares fitting the data to sets of model structure factors *F(H)*, each corresponding to a *d *repeat of D.

F(h)∑H=0HmaxF(H)[sin⁡(πDh/d−πH)/(πDh/d−πH)]     (1)
 MathType@MTEF@5@5@+=feaafiart1ev1aaatCvAUfKttLearuWrP9MDH5MBPbIqV92AaeXatLxBI9gBaebbnrfifHhDYfgasaacH8akY=wiFfYdH8Gipec8Eeeu0xXdbba9frFj0=OqFfea0dXdd9vqai=hGuQ8kuc9pgc9s8qqaq=dirpe0xb9q8qiLsFr0=vr0=vr0dc8meaabaqaciaacaGaaeqabaqabeGadaaakeaacqWGgbGrcqGGOaakcqWGObaAcqGGPaqkdaaeWbqaaiabdAeagjabcIcaOiabdIeaijabcMcaPiabcUfaBjGbcohaZjabcMgaPjabc6gaUjabcIcaOGGaciab=b8aWjabdseaejabdIgaOjabc+caViabdsgaKjabgkHiTiab=b8aWjabdIeaijabcMcaPiabc+caViabcIcaOiab=b8aWjabdseaejabdIgaOjabc+caViabdsgaKjabgkHiTiab=b8aWjabdIeaijabcMcaPiabc2faDbWcbaGaemisaGKaeyypa0JaeGimaadabaGaemisaG0aaSbaaWqaaGqaciab+1gaTjab+fgaHjab+Hha4bqabaaaniabggHiLdGccaWLjaGaaCzcamaabmaabaGaeGymaedacaGLOaGaayzkaaaaaa@625D@

The form factors and scattering length density profiles (see Eq. 3 below) were scaled to match *ρ*(0), the average scattering length density of the unit cell, defined as a bilayer (two monolayers):

*ρ*(0) = 2/*d*(*b*_*lip *_+ *b*_*prot*_)     (2)

where *b*_*lip *_and *b*_*prot *_represent the coherent scattering lengths of an "average" lipid molecule and 1 mol% peptide (where present), respectively. Note that *F*(0) corresponds to *ρ*(0)*d*/2. The scattering lengths of the component atoms were obtained from standard tables. Finally, the phased and scaled structure factors and corresponding Bragg spacings were used to reconstruct trans-bilayer coherent neutron scattering density profiles by Fourier synthesis:

ρ(x)=ρ(0)+2/d∑H=1HmaxF(h)cos⁡(2πxh/d)     (3)
 MathType@MTEF@5@5@+=feaafiart1ev1aaatCvAUfKttLearuWrP9MDH5MBPbIqV92AaeXatLxBI9gBaebbnrfifHhDYfgasaacH8akY=wiFfYdH8Gipec8Eeeu0xXdbba9frFj0=OqFfea0dXdd9vqai=hGuQ8kuc9pgc9s8qqaq=dirpe0xb9q8qiLsFr0=vr0=vr0dc8meaabaqaciaacaGaaeqabaqabeGadaaakeaaiiGacqWFbpGCcqGGOaakcqWG4baEcqGGPaqkcqGH9aqpcqWFbpGCcqGGOaakcqaIWaamcqGGPaqkcqGHRaWkcqaIYaGmcqGGVaWlcqWGKbazdaaeWbqaaiabdAeagjabcIcaOiabdIgaOjabcMcaPiGbcogaJjabc+gaVjabcohaZjabcIcaOiabikdaYiab=b8aWjabdIha4jabdIgaOjabc+caViabdsgaKjabcMcaPaWcbaGaemisaGKaeyypa0JaeGymaedabaGaemisaG0aaSbaaWqaaGqaciab+1gaTjab+fgaHjab+Hha4bqabaaaniabggHiLdGccaWLjaGaaCzcamaabmaabaGaeG4mamdacaGLOaGaayzkaaaaaa@5B38@

Although the composition of the unit cell is known, its volume (S × *d*, where S is the "average" area per lipid) is not. However, multiplying both sides of equation [3] by S introduced a "per lipid" scale, and provided convenient dimensionless units of "scattering density" [45]. The errors of the profiles were calculated from.

Δρ(x)=2t/d[∑H=0Hmax(ΔF(h))2cos⁡2(2πxh/d)]0.5     (4)
 MathType@MTEF@5@5@+=feaafiart1ev1aaatCvAUfKttLearuWrP9MDH5MBPbIqV92AaeXatLxBI9gBaebbnrfifHhDYfgasaacH8akY=wiFfYdH8Gipec8Eeeu0xXdbba9frFj0=OqFfea0dXdd9vqai=hGuQ8kuc9pgc9s8qqaq=dirpe0xb9q8qiLsFr0=vr0=vr0dc8meaabaqaciaacaGaaeqabaqabeGadaaakeaacqqHuoariiGacqWFbpGCcqGGOaakcqWG4baEcqGGPaqkcqGH9aqpcqaIYaGmcqWG0baDcqGGVaWlcqWGKbazcqGGBbWwdaaeWbqaaiabcIcaOiabfs5aejabdAeagjabcIcaOiabdIgaOjabcMcaPiabcMcaPmaaCaaaleqabaGaeGOmaidaaOGagi4yamMaei4Ba8Maei4Cam3aaWbaaSqabeaacqaIYaGmaaGccqGGOaakcqaIYaGmcqWFapaCcqWG4baEcqWGObaAcqGGVaWlcqWGKbazcqGGPaqkcqGGDbqxdaahaaWcbeqaaiabicdaWiabc6caUiabiwda1aaaaeaacqWGibascqGH9aqpcqaIWaamaeaacqWGibasdaWgaaadbaacbiGae4xBa0Mae4xyaeMae4hEaGhabeaaa0GaeyyeIuoakiaaxMaacaWLjaWaaeWaaeaacqaI0aanaiaawIcacaGLPaaaaaa@63A8@

where Δ*ρ*(*x*) is the error in *x *at a confidence limit of 95% (t = 1.96), and *Δ*F(*h*) are the (independent) form factor errors from the fitting process described by equation [1].

### Membrane lipid order

Multilamellar lipid (or proteolipid) vesicles were prepared exactly as described for membrane neutron diffraction, apart from the addition of 5 mol% laurdan. Measurements were carried out at 25°C (at a lipid concentration of 50 μM) in a Shimadzu RF-5000 spectrofluorophotometer, using the protocol previously described [20], and following excitation at 350 nm generalised polarisation (GP) was calculated from the relationship: GP = (*I*_435 _- *I*_500_)/(*I*_435 _+ *I*_500_), where *I*_435 _and *I*_500 _are the steady-state emission intensities at 435 nm and 500 nm, respectively.

### Statistical analysis

Differences were assessed by t-testing, and were considered significant if P < 0.05.

## Abbreviations

Aβ, amyloid-β protein; AD, Alzheimer's Disease; Chol, cholesterol; GP, generalised polarisation; POPC, palmitoyl-oleoyl phosphatidylcholine; SLD, scattering length density.

## Authors' contributions

RHA, JPB, KCB & TAH designed the research; RHA, TAH, JPB & TH carried out the experiments; RHA, TAH & JPB analysed the data; RHA wrote the paper. All the authors read and approved the final manuscript.

## Supplementary Material

Additional file 1 Structure factors obtained in 8.06 mol% D_2_O. Q is the peak position in reciprocal space, and F(*h*) and *Δ*F(*h*) are the form factors and their errors, respectively. Click here for file

Additional file 2 Oligomeric profile of Aβ(1–42). Six independent oligomer preparations (O_1_-O_6_) were subjected to non-reducing 16.5% (w/v) Tris-Tricine SDS-PAGE with silver staining, as described by Lambert et al. [4], using Bio-Rad Precision Plus unstained markers supplemented with human insulin (lanes labelled "S" in each experiment). The first gel includes a sample of monomeric Aβ(1–42) prior to oligomerisation, which was carried out as described [4]. Three major molecular species of Aβ(1–42) – monomer, trimer and tetramer – are arrowed to the right of the gels. The overall grey background arises from the silver staining procedure (PlusOne Silver Staining Kit, Amersham Pharmacia Biotech). Click here for file
