# Supplementary material for: Autoinsertion of soluble oligomers of Alzheimer's Aβ(1–42) peptide into cholesterol-containing membranes is accompanied by relocation of the sterol towards the bilayer surface
Source: BMC Struct Biol. 2006 Oct 19;6:21. doi: 10.1186/1472-6807-6-21 (PMC1657013; doi:10.1186/1472-6807-6-21)
Supplement: Additional file 1 — Structure factors obtained in 8.06 mol% D2O. Q is the peak position in reciprocal space, and F(h) and ΔF(h) are the form factors and their errors, respectively. [file 1472-6807-6-21-S1.PDF]

Unlabelled lipid-only membranes:

order	Q ($Å^{-1}$)	scaled F(h)	ΔF(h)
0	0.00	2.96690	---
1	0.017544	-5.84336	0.178
2	0.035088	-1.63372	0.175
3	0.052632	1.379564	0.172
4	0.070175	-0.94346	0.172
5	0.087700	0.138000	0.450

Unlabelled lipids + Aβ:

order	Q ($Å^{-1}$)	scaled F(h)	ΔF(h)
0	0.00	3.63110	---
1	0.017544	-5.38797	0.115
2	0.035088	-2.57288	0.115
3	0.052632	1.34386	0.115
4	0.070175	-0.756030	0.114
5	0.087700	0.108210	0.219

Labelled Chol:

order	Q ($Å^{-1}$)	scaled F(h)	ΔF(h)
0	0.00	5.00200	---
1	0.017544	-5.65814	0.469
2	0.035088	-3.87085	0.427
3	0.052632	2.03267	0.384
4	0.070175	-0.562180	0.329
5	0.087700	0.148915	0.252

Labelled Chol + Aβ:

order	Q ($Å^{-1}$)	scaled F(h)	ΔF(h)
0	0.00	5.57950	---
1	0.017544	-6.25271	0.314
2	0.035088	-3.88787	0.314
3	0.052632	2.16475	0.314
4	0.070175	-0.371390	0.314
5	0.087700	0.651450	0.320

Labelled POPC:

order	Q ($Å^{-1}$)	scaled F(h)	ΔF(h)
0	0.00	26.8286	---
1	0.017544	7.48275	0.097
2	0.035088	-2.71316	0.095
3	0.052632	-0.166690	0.094
4	0.070175	-0.0149900	0.116
5	0.087700	0.114380	0.120

Labelled POPC + Aβ:

order	Q ($Å^{-1}$)	scaled F(h)	ΔF(h)
0	0.00	26.4985	---
1	0.017544	7.91200	0.390
2	0.035088	-2.68842	0.372
3	0.052632	0.197830	0.441
4	0.070175	0.0797650	0.650
5	0.087700	0.016000	0.608
