# Supplementary figures and images for: Autoinsertion of soluble oligomers of Alzheimer's Aβ(1–42) peptide into cholesterol-containing membranes is accompanied by relocation of the sterol towards the bilayer surface
Source: BMC Struct Biol. 2006 Oct 19;6:21. doi: 10.1186/1472-6807-6-21 (PMC1657013; doi:10.1186/1472-6807-6-21)

Mr (K)

samples

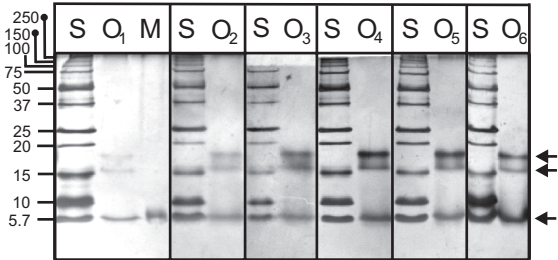

Supplement: Additional file 2 — Oligomeric profile of Aβ(1–42). Six independent oligomer preparations (O1-O6) were subjected to non-reducing 16.5% (w/v) Tris-Tricine SDS-PAGE with silver staining, as described by Lambert et al. [4], using Bio-Rad Precision Plus unstained markers supplemented with human insulin (lanes labelled "S" in each experiment). The first gel includes a sample of monomeric Aβ(1–42) prior to oligomerisation, which was carried out as described [4]. Three major molecular species of Aβ(1–42) – monomer, trimer and tetramer – are arrowed to the right of the gels. The overall grey background arises from the silver staining procedure (PlusOne Silver Staining Kit, Amersham Pharmacia Biotech). [file 1472-6807-6-21-S2.pdf]
